# An RSM-Based Investigation on the Process–Performance Correlation and Microstructural Evolution of Friction Stir Welded 7055 Al/2195 Al-Li Dissimilar T-Joints

**DOI:** 10.3390/ma19061260

**Published:** 2026-03-23

**Authors:** Binbin Lin, Yanjie Han, Duquan Zuo, Nannan Wang, Yuanxiu Zhang, Haoran Fu, Chong Gao

**Affiliations:** 1 Sichuan Province Engineering Technology Research Center of General Aircraft Maintenance, Civil Aviation Flight University of China, 46 Nanchang Road, Guanghan 618307, China; linbinbin@cafuc.edu.cn (B.L.); 18726537934@163.com (Y.H.); 17360157267@163.com (Y.Z.); fuhaoran74@gmail.com (H.F.); 2 College of Aviation Engineering, Civil Aviation Flight University of China, 46 Nanchang Road, Guanghan 618307, China; 3 Luoyang College, Civil Aviation Flight University of China, Luoyang 471000, China; 15896555218@163.com; 4 School of Engineering, University of Tokyo, 7-3-1 Hongo, Bunkyo-ku, Tokyo 113-8656, Japan; gaochong@fc.ritsumei.ac.jp

**Keywords:** friction stir welding, T-joints, response surface methodology, mechanical properties, microstructure evolution, dynamic recrystallization

## Abstract

Friction stir welding (FSW) is a key technology for manufacturing T-shaped thin-walled structures and avoiding fusion welding defects. However, the quantitative relationship between its process parameters and the microstructure properties of the joint remains unclear. To address this, this study established regression models via response surface methodology (RSM) relating rotational speed (*w*), welding speed (*v*), and plunge depth (*h*) to the mechanical properties of T-joints. The optimal process parameters (400 rpm, 60 mm/min, 0.21 mm) were determined, under which the ultimate tensile strength (UTS) and weld nugget hardness (WNH) of the joint reached 74.1% (377 MPa) and 94.4% (153 Hv) of the base materials (BM) respectively, with *v* showing the most significant influence on joint mechanical properties. Microstructural observations revealed that from the BM to the stirring zone (SZ), the grains underwent a continuous evolution from coarsening, partial recrystallization to complete dynamic recrystallization (DRX). In the SZ, due to severe plastic deformation and high heat input, the continuous dynamic recrystallization (CDRX) was the dominant mechanism, and the grain was significantly refined. The heat input in the thermomechanical affected zone (TMAZ) is relatively low, mainly geometric dynamic recrystallization (GDRX). DRX-driven grain refinement was the primary strengthening factor in the joint, with hardness closely related to grain size. However, thermal cycling induced softening in the heat-affected zone (HAZ) and promoted the precipitation of brittle compounds such as Al_3_Mg_2_ and MgZn_2_, which caused crack initiation exhibiting intergranular brittle fracture. Subsequently, under stress drive, it extends to SZ, mainly characterized by ductile fracture.

## 1. Introduction

7xxx series aluminum alloys and the lower-density third-generation aluminum–lithium alloys have become ideal materials for integrated thin-walled panel structures in the aerospace industry due to their excellent specific strength and specific stiffness [1,2,3]. The T-joint lap configuration is a typical connection form in such structures, effectively enhancing the stiffness and bending resistance of skins [4], and is widely used in critical components such as fuselages, wing ribs, and bulkheads. However, when traditional fusion welding techniques such as arc welding or laser welding are employed for T-joint connections, defects such as hot cracking, porosity, lithium element burnout, and significant post-weld deformation of the plate are prone to occur. In contrast, friction stir welding (FSW), as an emerging solid-state joining technology [5,6,7,8], not only effectively avoids the metallurgical defects associated with traditional fusion welding but also significantly refines the grain structure in the weld zone. Additionally, it overcomes the drawbacks of conventional riveting, such as large weight and low efficiency [9,10], providing a highly promising technical pathway for achieving high-performance lightweight structural connections in the aerospace field.

It is noteworthy that FSW involves multiple process variables that directly influence joint performance, with key parameters such as rotational speed (*w*), welding speed (*v*), and plunge depth (*h*) being the most critical [11,12,13,14,15,16,17,18,19]. Generally speaking, a higher *w* combined with a lower *v* increases the heat input, which helps to enhance material plastic flow in the weld zone and reduce defects, thereby improving weld quality [11,12,13,14,15]. Conversely, lower *w* and higher *v* result in insufficient heat input, causing defects such as lack of penetration and voids, which impair the overall performance of the joint [16,17,18]. Besides *w* and *v*, *h* also significantly affects the weld formation of the joint. An excessively small *h* leads to insufficient heat input, making it difficult to achieve a high-quality joint, whereas an excessively large *h* tends to cause excessive weld depression, resulting in stress concentration and reduced joint performance [19]. Given the significant influence of the above-mentioned process parameters on weld quality, systematic optimization of them is essential to obtain the best joint performance and achieve high-quality welding.

Previous studies have shown that systematic experimental design methods combined with specimen testing within a specific process range can effectively determine the optimal combination of FSW process parameters, thereby obtaining welded joints with better comprehensive performance [20,21]. Chen et al. [22] established a regression model between welding process parameters and mechanical properties based on the orthogonal experimental method, finding that when *w* = 400 rpm, *v* = 70 mm/min, and *h* = 0.15 mm, the tensile strength of butt joints increased by 46.15%. Lakache and Muchhadiya et al. [23,24] optimized butt joints using an L16 Taguchi design, achieving joint efficiency reaching 95.29% of the base material. For dissimilar material butt joints, Abdelhady and Dugar et al. [25,26] employed multi-objective optimization design methods and gray relational analysis (GRA) to evaluate the comprehensive performance of tensile strength and hardness. The response surface methodology (RSM) has also been widely used to improve the mechanical properties and microstructure of FSW joints due to its high efficiency [27]. Among them, the Central Composite Design method (CCD) is a classic second-order experimental design method, which was used by Kumar et al. [28] to construct a mathematical prediction model. It was found that the tensile strength of the dissimilar material butt joint could reach 82% of the base material under the optimal parameters. In summary, current FSW process parameter optimization mainly focuses on the macroscopic properties of similar or dissimilar material butt joints, while comprehensive research on both the macroscopic and microscopic performance of dissimilar FSW T-joints is still very rare [29]. This is particularly evident in the field of dissimilar welding involving newer alloys such as 7055-T6 Al and 2195-T6 Al-Li alloys. Furthermore, within RSM, the Box–Behnken design (BBD) [30] shows significant application advantages compared to the CCD method, as it can substantially reduce the number of experiments and lower research costs while maintaining model accuracy.

For this purpose, in this paper, for the 7055-T6/2195-T6 dissimilar alloy FSW-T-joint, the BBD experimental design method is adopted to construct its mechanical property prediction model, and the significant influence of *w*, *v*, and *h* on the weld nugget hardness (WNH) distribution and ultimate tensile strength (UTS) is systematically analyzed. Furthermore, the optimal combination of process parameters is determined to achieve the comprehensive performance optimization of the T-joint. Meanwhile, by integrating microstructural characterization, DRX grain statistics, and fracture morphology analysis, the intrinsic correlation mechanism among “process parameters-microstructural evolution-DRX behavior-mechanical response” is thoroughly elucidated, providing a theoretical basis and technical support for the engineering application of dissimilar alloy T-joints in lightweight, high-performance thin-walled structures in the aerospace field.

## 2. Materials and Methods

**Figure 1:**
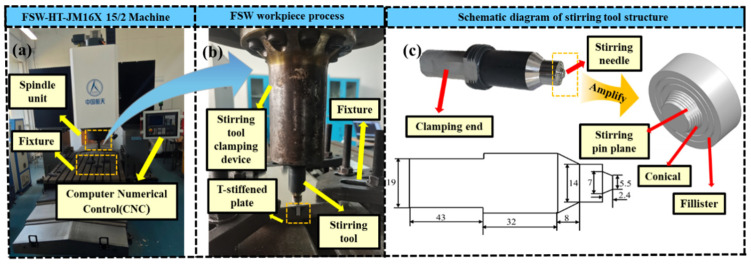
Welding equipment structure and triangular cone-shaped threaded stirring tool: (**a**) FSW-LM-L10 gantry friction stir welding machine; (**b**) Welding process; (**c**) Schematic diagram of the dimensions and geometric structure of the stirring tool.

When welding ultra-thin-walled plate structures (0.5–1.5 mm) using FSW technology, problems such as insufficient heat input, thinning of the workpiece, microscopic defects, difficult clamping, and unbalanced distribution of residual stress are often encountered [31,32,33]. To address the above-mentioned technological problems, in this study, the FSW-LM-L10 type gantry CNC friction stir welding machine was used to perform T-type welding on the 7055-T6 Al alloy (skin) with dimensions of 200 × 100 × 2 mm and 2195-T6 Al-Li alloy (stringer) with dimensions of 200 × 13 × 5 mm. The welding process is illustrated in Figure 1a,b, and the chemical compositions and room-temperature mechanical properties are detailed in Table 1 and Table 2. The stirring tool was made of H13 high-carbon steel, with a shoulder diameter of 14 mm. The root diameter, end diameter and length of the stirring pin are 7 mm, 5.5 mm and 2.4 mm, respectively. The stirring pin featured a triangular tapered threaded structure, and the side is provided with planes at intervals of 120° to enhance material flow capability. Its geometric dimensions are shown in Figure 1c. This paper focuses on the influence of *w*, *v* and *h* on the mechanical properties and microstructure of weld seams. Therefore, the tool geometry, dimensions, and tool tilt angle (2°) are set as constants.

To prepare welded joints with high strength and free from macro- and micro-defects, this study determined the feasible process range of 7055-T6/2195-T6 dissimilar alloy FSW connections through a series of preliminary experiments. This range is divided into three levels: lower limit (−1), baseline (0), and upper limit (+1), with specific thresholds listed in Table 3. The BBD within the RSM is employed to optimize the FSW process parameters, aiming to simultaneously enhance the UTS and WNH of the joints. Based on this method, 17 sets of process parameter combinations are constructed, as detailed in Table 4.

**Figure 2:**
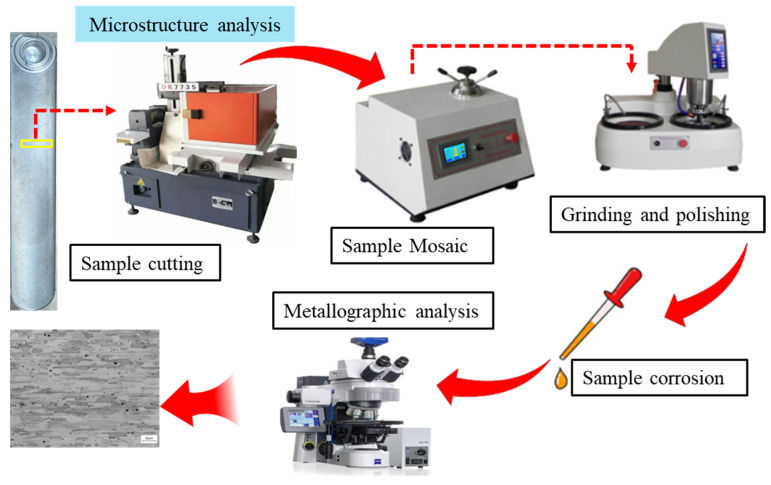
Sample preparation and microstructure observation process.

Perpendicular to the weld seam, metallographic samples were extracted from the welded joint by wire electrical discharge machining (EDM). After mechanical grinding and polishing, the samples were subjected to etching treatment using Keller’s reagent (1 mL HF + 1.5 mL HCl + 2.5 mL HNO_3_ + 9 mL H_2_O). Subsequently, the microstructures of the etched samples were observed and analyzed using a metallographic microscope (Germany-ZEISS-Axio Imager 2, German Zeiss Company, Oberkochen, Germany). The sample preparation and observation procedure is illustrated in Figure 2. For further microstructural analysis, electron backscatter diffraction (EBSD) was employed to characterize unetched samples, with a focus on investigating the crystal orientation distribution and grain boundary evolution.

**Figure 3:**
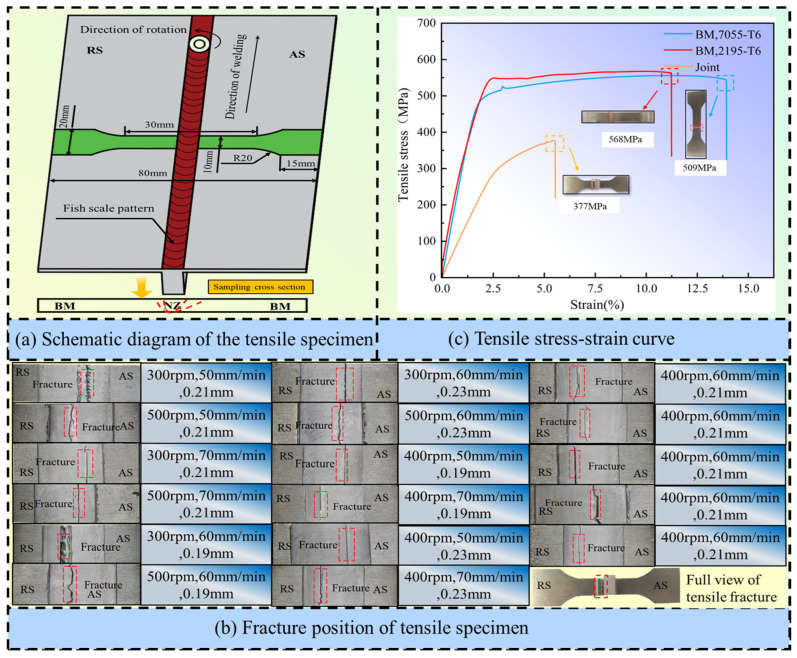
Process of tensile test under different parameters: (**a**) Schematic diagram of the size of the tensile specimen, (**b**) tensile stress–strain curve and (**c**) fracture position of the tensile specimen.

Tensile specimens were prepared in accordance with the Chinese national standard GB/T 228.1-2021, with their axes perpendicular to the weld direction. The specific sampling location and geometric dimensions are shown in Figure 3a. Uniaxial tensile tests were conducted perpendicular to the weld direction using an electronic universal testing machine (Instron 68FM-100, InstronTest Equipment Trading Co., Ltd., Shanghai, China) to obtain the engineering stress–strain curves of the joints under different parameters (Figure 3b), and the obtained UTS values were recorded in Table 4. The typical fracture morphologies of specimens under different parameters are shown in Figure 3c. The fracture morphology was examined using a scanning electron microscope (SEM: ZEISS Sigma 360, Shenzhen Huapeng General Technology Co., Ltd., Shenzhen, Guangdong, China) to analyze the fracture mechanisms. Based on the aforementioned polished metallographic specimens, Vickers hardness testing was conducted using the ZHV-1DT microhardness tester, Beijing Zhongyan Instrument Co., Ltd., Beijing, China. The tests were carried out in both the normal (ND) and transverse (TD) directions, with a load of 100 g and a holding time of 10 s. Each measurement was repeated three times, and the average value was taken. The obtained hardness values are also recorded in Table 4.

## 3. Experimental Results and Discussion

### 3.1. Joint Defect Analysis

**Figure 4:**
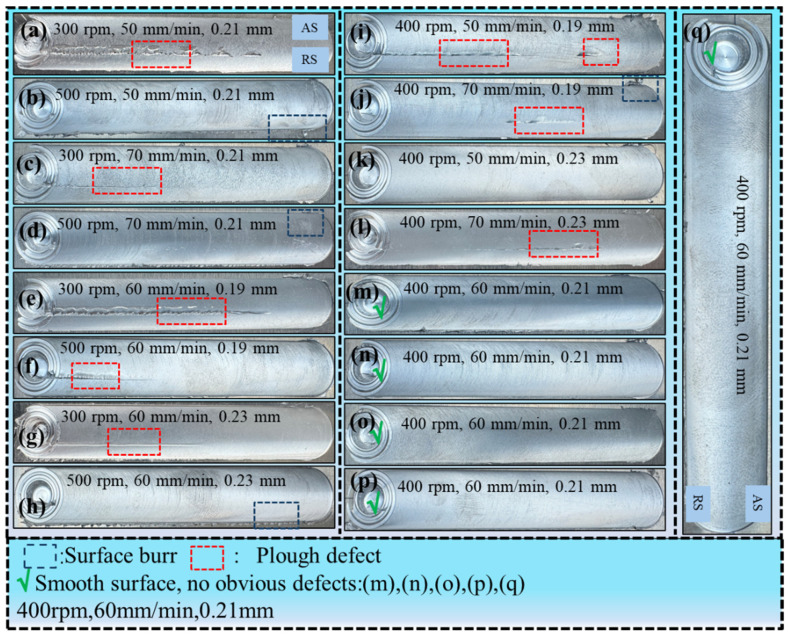
Macroscopic morphology of welds under various parameters.

The surface morphology of the welded T-joints is shown in Figure 4, clearly revealing the significant influence of welding process parameters on weld formation and defect evolution. As observed in Figure 4a,c,e–g,i,j,l), groove defects occurred in experimental groups 1, 3, 5, 6, 7, 9, 10, and 12. The mechanisms for defect formation varied depending on the combination of process parameters. The occurrence of this defect in experimental groups 1, 3, 5, and 7 is due to the low *w*, resulting in insufficient input of frictional heat. The flow resistance of plastic metals increases, and it is difficult for plastic materials to fill the cavities. For groups 6, 9, and 10, the groove defects resulted from an excessively small *h*, which resulted in insufficient pressure between the tool shoulder and the workpiece, preventing effective cavity filling by the plasticized material. The defect in group 12 was attributed to an excessively high *v*, which reduced the heat input per unit length, accelerated the material cooling rate, and caused transient cavities to close inadequately. In contrast, the joint surface morphology of experimental groups 2, 4, 8, and 11 exhibited significant flash, as shown in Figure 4b,d,h,k. Among them, the flash in experimental groups 2, 4, and 8 was due to excessive heat input, which generated a large amount of plasticized material that was thrown out during high-speed rotation. In experimental group 11, flash resulted from excessive axial pressure forcibly extruding the plasticized material. Notably, the joint surface morphology corresponding to experimental groups 13, 14, 15, 16, and 17 (Figure 4m–q) was good. This indicates that smooth welds without macroscopic defects can be obtained by optimizing and selecting the appropriate combination of process parameters, further demonstrating that the appropriate process parameters play a decisive role in the macroscopic forming quality of FSW joints.

### 3.2. Establishment of a Mathematical Model Based on RSM

Based on the input parameters (*w*, *v*, *h*) listed in Table 3 and the corresponding mechanical performance outputs of the joints (UTS and WNH, see Table 4), this study employed RSM [34] to develop a process parameter mathematical model (PMM). The model quantitatively characterizes the nonlinear functional relationships between the process parameters and the joint performance indicators (UTS and WNH). Based on this, determine the optimal combination of process parameters. Multi-objective modeling was performed using Design Expert software, 13.0. Through statistical methods such as sequential lack-of-fit tests, F-tests, and analysis of variance (ANOVA), the fit degree, term significance and interaction effect of the model were systematically evaluated to verify the reliability and accuracy of the model. The established general expression of PMM is shown in Formula (1) [35].

*N* = *f* (*w*, *v*, *h*) (1)

To deeply analyze the mathematical structure of the response function defined by Equation (1), this study employs a high-order polynomial regression model to analyze the influence of process parameters (*w*, *v*, *h*) on the target response. Given that a first-order model is insufficient to adequately capture the nonlinear characteristics within the parameter space, a second-order response surface model is specifically introduced. This enables a high-precision approximation of the response targets by the process parameters within the selected ranges. This model can be uniformly expressed as:

(2) $$N=b_{0}+\sum b_{i}x_{i}+\sum b_{\mathrm{ii}}x_{i}^{2}+\sum b_{\mathrm{ij}}x_{i}x_{j}$$

In the formula, *N* represents the predicted response; *x* is the encoded independent variable; *b*_0_ is the model intercept; and *b_i_*, *b_ij_*, and *b_ii_* correspond to the regression coefficients for the linear, interaction, and quadratic effects, respectively. Expanding Equation (2) specifically as:

(3) $$N=\left\{b_{0}+b_{1}\left(w\right)+b_{2}\left(v\right)b_{3}\left(h\right)+b_{12}\left(w\;v\right)+b_{13}\left(w\;h\right)+b_{23}\left(v\;h\right)+b_{11}{\left(w\right)}^{2}+b_{22}{\left(v\right)}^{2}+b_{33}{\left(h\right)}^{2}\right\}$$

where *w*, *v*, and *h* represent the coded independent variables. Based on the Design Expert platform, the significance test of each coefficient of the model was conducted at the 95% confidence level, and the insignificant items were eliminated to improve the prediction accuracy of the model. Ultimately, a PMM with only significant terms retained is obtained, and its mathematical model can be expressed as follows:

(4) $$\left\{\begin{array}{c} UTS\;=\;369.2\;+\;15w\;-\;28.25v\;+\;18.25h\;-\;72wv\;-\;6wh\;-\;16vh\;-\;89.6w^{2}\;-\;66.1v^{2}\;-\;113.1h^{2}\\ WNH\;=\;152.42\;+\;2.28w\;-\;5.05v\;+\;3.93h\;-\;15.35wv\;-\;1.5wh\;-\;3.75vh\;-\;14.16w^{2}\;-\;8.76v^{2}\;-\;17.26h^{2} \end{array}\right.$$

#### Evaluating the Feasibility of the Generated Parametric Mathematical Model

**Figure 5:**
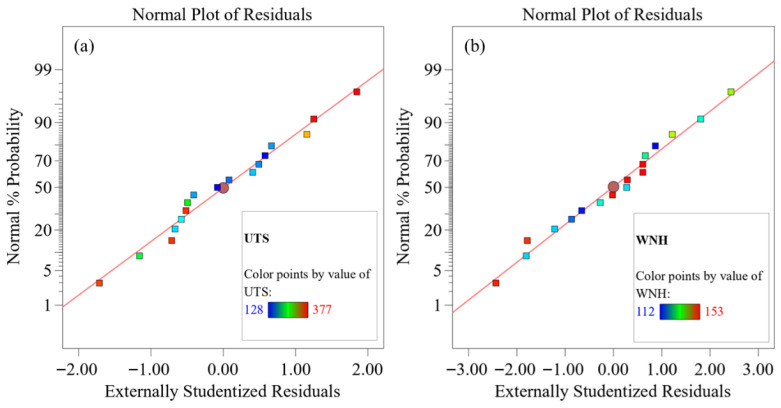
Normal probability plot of residuals for (**a**) UTS and (**b**) WNH.

ANOVA, as a statistical method for evaluating the significance of mean differences among multiple groups of samples, can effectively reveal the relationship between dependent and independent variables. ANOVA typically includes the F-test and the P-test. The P-test is primarily used to determine whether an individual hypothesis holds, with the criterion being: a *p*-value less than 0.05 indicates that the model term is significant, and the smaller *p*-value corresponds to higher significance. The F-test is suitable for multi-parameter statistical models and serves as an effective method for determining whether design parameters exert a substantial influence on the target attributes, with the criterion being: a higher F-value indicates a greater influence of that parameter on the response value. Therefore, to investigate the significance of the influence of *w*, *v*, and *h* on the UTS and WNH of T-joints, the F and P criteria from ANOVA were employed to statistically test the experimental results. The ANOVA data for UTS and WNH are presented in Table 5 and Table 6, respectively. As shown in the tables, the *p*-values for both models are less than 0.0001, indicating that the constructed response surface models are highly significant. Meanwhile, the F-values of Factor A (*w*), Factor B (*v*), and Factor C (*h*) in Table 5 and Table 6 are (48.5, 172.02, and 71.79) and (46.25, 196.49, and 129.52), respectively. Among them, the F values of factors B (*v*) and C (*h*) are relatively high, indicating that they are the dominant factors affecting UTS and WNH. The relatively lower F-value for Factor A (*w*) suggests its influence on UTS and WNH is weaker than that of Factors B and C, but it is not completely unaffected. In addition, the R^2^ values, adjusted R^2^ values, and predicted R^2^ values of the model all exceed 0.9. Although the R^2^ values in Table 5 and Table 6 reach 0.99, the differences between the predicted R^2^ and adjusted R^2^ in these tables are less than 0.02, indicating that the model does not suffer from overfitting and that its predictive capability aligns well with its fitting ability. Furthermore, the adequate precision values in Table 5 and Table 6 are 51.78 and 52.72, respectively, both substantially greater than 4, demonstrating that the model exhibits strong signal strength, minimal noise interference, sufficient resolution, and a high signal-to-noise ratio. Meanwhile, the lack-of-fit terms of both models are not significant, further confirming the good fit of the models to the experimental data. Moreover, as can be seen from the normal probability plots of residuals in Figure 5a,b, the residual points are essentially distributed along the theoretical straight line, and the errors are normally distributed, which once again verifies the reliability and accuracy of the prediction model.

**Figure 6:**
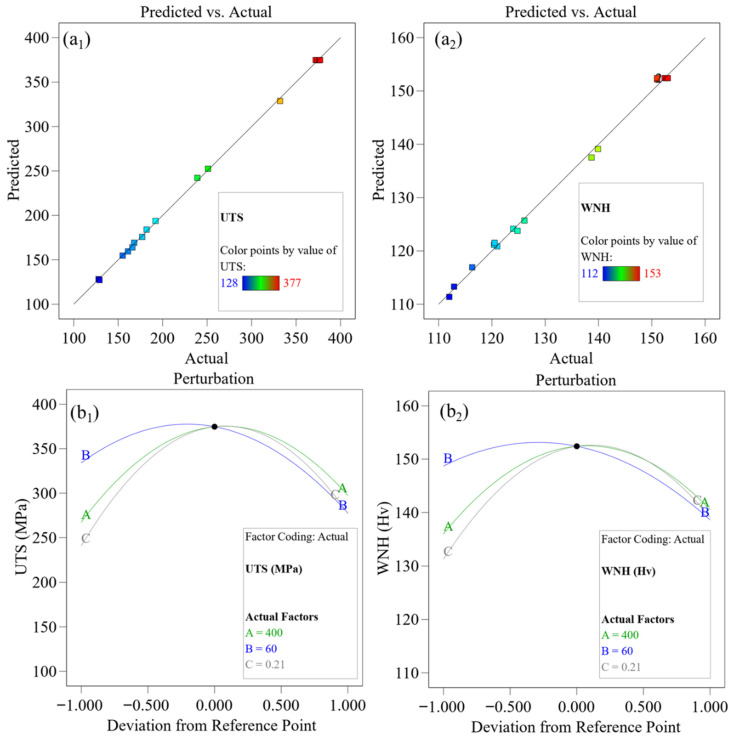
Perturbation curves under the fitting of actual and predicted values and process parameters: Fitting of actual and predicted values (**a_1_**) UTS and (**a_2_**) WNH, perturbation curves (**b_1_**) UTS and (**b_2_**) WNH.

To validate the applicability of the developed models in the actual welding process, an accuracy test was conducted on the PMMs for the UTS and WNH of the T-joint, as shown in Figure 6(a_1_,a_2_). The figure shows that the predicted values from the response surface model and the actual measured values are approximately distributed along the *y* = *x* line, indicating high predictive accuracy of the model and good consistency between theoretical calculations and experimental results. Figure 6(b_1_,b_2_) present the perturbation curves of the three process parameters, which visually reflect the influence of parameter variations within the design space on the response values. The absolute value of the curve slope reflects the sensitivity of the joint UTS and WNH to parameter changes. The larger the absolute value, the more significant the influence of this parameter on the response value [36]. As can be seen from Figure 6(b_1_,b_2_), the absolute value of the slope of curve B is the largest, followed by curve C, and the slope of curve A is the smallest. This demonstrates that *v* has the most significant influence on UTS and WNH, followed by *h*, while *w* has the weakest influence. This observation is consistent with the influence patterns revealed by the F-values in the previous ANOVA.

### 3.3. Multi-Objective Optimization and Verification Based on Response Surface Model

**Figure 7:**
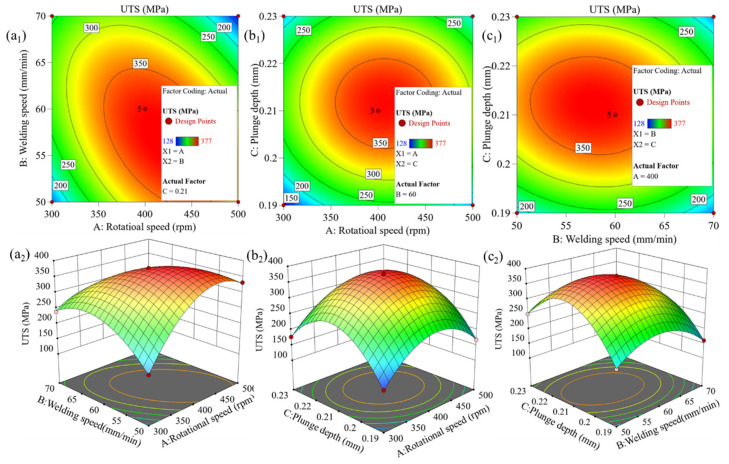
Contour and 3D surface plots of UTS showing the interaction between rotational speed and welding speed (**a_1_**,**a_2_**), rotational speed and plunge depth (**b_1_**,**b_2_**), and welding speed and plunge depth (**c_1_**,**c_2_**).

**Figure 8:**
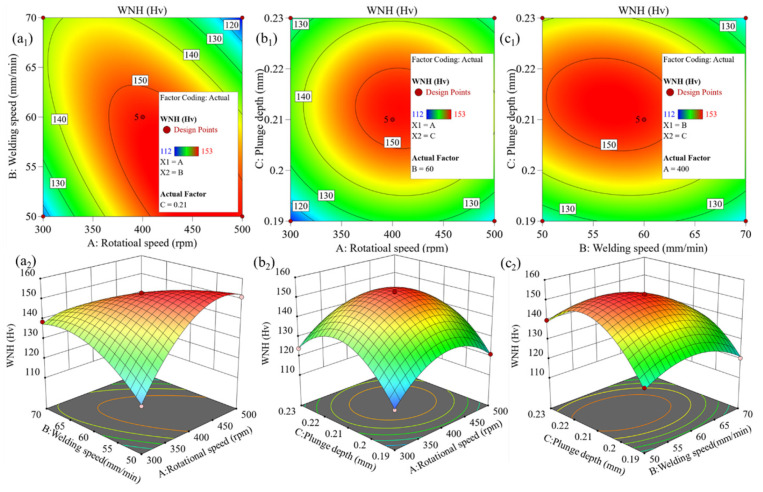
Contour and 3D surface plots of WNH showing the interaction between rotational speed and welding speed (**a_1_**,**a_2_**), rotational speed and plunge depth (**b_1_**,**b_2_**), and welding speed and plunge depth (**c_1_**,**c_2_**).

Based on the established PMM (see Equation (4)), the influence of the interactions among *w*, *v*, and *h* on UTS and WNH of the joint was systematically investigated by generating contour maps and three-dimensional response surface plots. Generally, the steeper the response surface or the closer the contour lines are to an ellipse, the stronger the interaction between the two factors. Figure 7 and Figure 8 present the contour maps and 3D response surface plots for UTS and WNH, respectively. Analysis reveals that for the two response values of UTS and WNH, the interaction between factors *w* and *v* is the strongest, followed by that between *v* and *h*, while the interaction between *w* and *h* is the weakest. By analyzing the contour maps and response surface plots, it was determined that among the 17 sets of experimental parameters studied, the joint performance reaches its optimum when the process parameters are *w* = 400 rpm, *v* = 60 mm/min, and *h* = 0.21 mm. At this combination, UTS and WNH achieve their maximum values of 377 MPa and 153 Hv, respectively, corresponding to 74.1% and 94.4% of the BM. Further analysis of the parameter influence patterns shows that both UTS and WNH increase with the rise in *w*, *v*, and *h* until a specific threshold is reached. This phenomenon is attributed to the sufficient heat input and plastic deformation within the appropriate parameter range, which promotes adequate material flow and effective bonding. However, when the parameter combination exceeds this optimal point, further increases in *w*, *v*, or *h* lead to a decline in UTS and WNH. This is due to excessive frictional heat causing over-softening or excessive deformation in the joint region. Table 7 presents three sets of predicted process parameter solutions based on different optimization criteria. The optimal prediction parameters of the model obtained based on the criterion of “maximum satisfaction” are (431 rpm, 57 mm/min, 0.21 mm), and the predicted UTS and WNH are 370 MPa and 153 Hv, respectively, which are in high agreement with the experimental optimal values (377 MPa, 153 Hv). Given that the experimental value is slightly superior in UTS, the FSW process parameters (400 rpm, 60 mm/min, 0.21 mm) are determined as the optimal choice.

### 3.4. Mechanical and Metallurgical Properties of T-Joints Under Optimal Parameters

#### 3.4.1. Microstructural Evolution in the Weld Zone of the T-Joint

**Figure 9:**
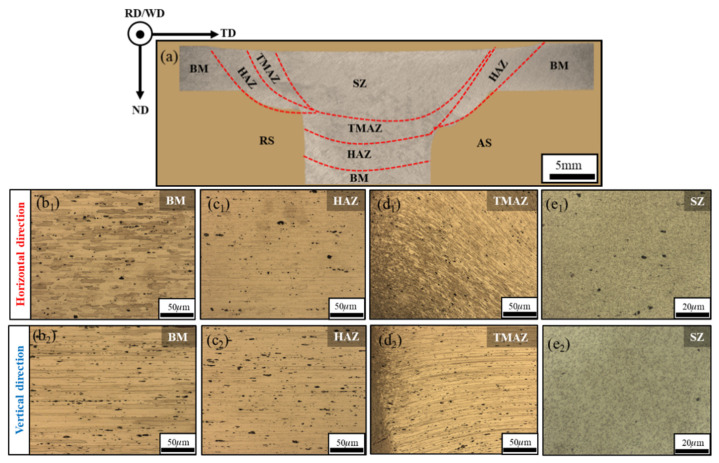
Optical micrographs of the T-joints comprising the weld zones: BM, HAZ, TMAZ and SZ. (**a**) Whole cross-sectional image covering all the weld zones, (**b_1_**–**e_1_**) and (**b_2_**–**e_2_**) high-magnification images of the BM, HAZ, TMAZ and SZ of horizontal and vertical directions.

The low-magnification metallographic microstructure of the T-joint cross-section (perpendicular to the WD plane) under the optimal parameter combination (400 rpm, 60 mm/min, 0.21 mm) is shown in Figure 9a, and the corresponding high-magnification images are provided in Figure 9(b_1_,b_2_,c_1_,c_2_,d_1_,d_2_,e_1_,e_2_). It can be observed from Figure 9a that when extending from BM to the center of SZ, the microstructure contrast shows a progressive brightening trend. This gradient results from the plastic deformation energy induced by the frictional stirring action of the tool, which increases gradually along the SZ direction. This distribution characteristic provides an important basis for defining the characteristic welding zones. In this study, the TMAZ and HAZ are collectively referred to as the transition zone. In the high-magnification metallographic images of the BM (Figure 9(b_1_,b_2_)), the BM grains exhibit a typical rolled aluminum-alloy structure, with grain diameter decreasing along the normal direction (ND) while being significantly elongated along the rolling direction (RD). In the adjacent HAZ (Figure 9(c_1_,c_2_)), although the grain morphology remains similar to that of the BM, it undergoes grain coarsening under the effect of welding thermal cycling, forming a relatively coarse microstructure. Observing TMAZ (Figure 9(d_1_,d_2_)), it was found that TMAZ has a dual-state structure where transverse and vertical (TD) stretched grains coexist with fine isometric recrystallized grains. This is due to the partial DRX of the grains under the effect of thermal–mechanical coupling. In the SZ (Figure 9(e_1_,e_2_)), a homogeneous fine equiaxed grain structure is formed due to significant plastic deformation and complete DRX under high temperature and intense shear. It is worth noting that in all microscopic regions, there are dispersed black second-phase particles (mainly intermetallic compounds). Their size increases gradually from the SZ to the TMAZ and further to the HAZ, while their distribution shifts from homogeneous dispersion in the SZ to localized segregation in the HAZ. This evolution behavior originates from the coupled thermal–mechanical effects during the welding process. Under the combined action of periodic heat input and plastic deformation, the second-phase particles undergo a dissolution-coarsening mechanism, while the grain recrystallization and growth processes lead to the reconstruction of the particle spatial distribution.

In summary, although there are significant differences in the BM composition in the horizontal and vertical directions, the grain evolution laws of the four characteristic regions show a high degree of consistency, successively undergoing coarsening, partial recrystallization, and complete recrystallization refinement processes from BM through HAZ, TMAZ to SZ. Meanwhile, the second-phase particles simultaneously exhibit a synergistic evolution feature of gradually decreasing size and increasing uniformity of distribution.

#### 3.4.2. Dynamic Recrystallization Behavior

**Figure 10:**
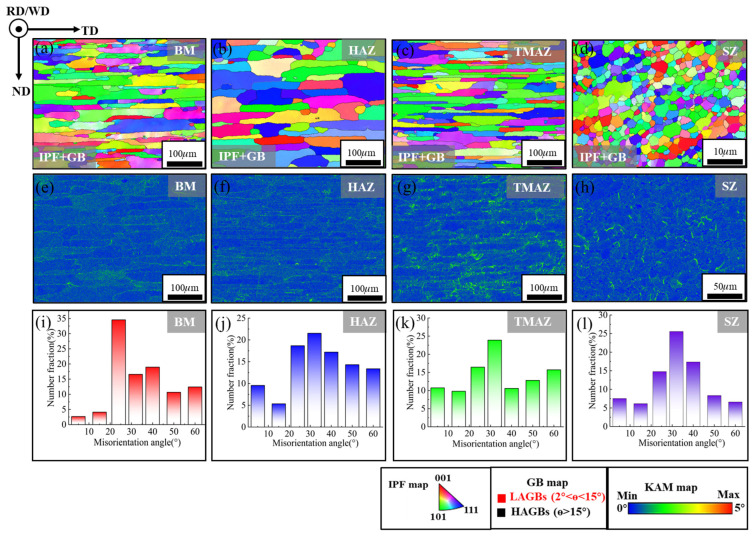
(**a**–**d**) Combined EBSD inverse pole figure (IPF) and grain boundary (GB) maps, (**e**–**h**) Kernel average misorientation (KAM) maps, and (**i**,**j**) misorientation angle distribution of BM (**a**,**e**,**i**), HAZ (**b**,**f**,**j**), TMAZ (**c**,**g**,**k**) and SZ (**d**,**h**,**l**) of FSWed T-joints.

**Figure 11:**
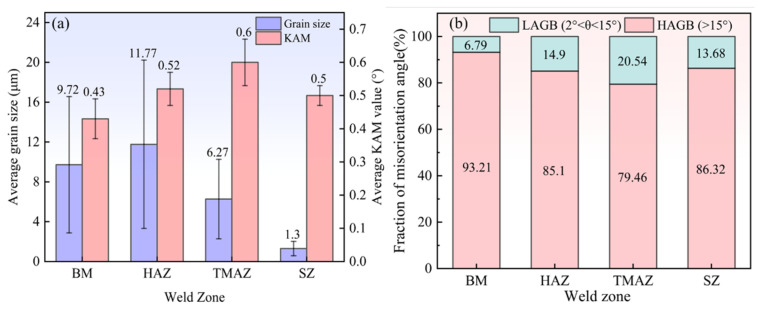
(**a**) Histogram of average grain size and average KAM value, and (**b**) fraction of high-angle and low-angle grain boundaries (HAGB and LAGB) in each weld zone.

Given that the BM, HAZ, TMAZ, and SZ of the T-joint exhibit consistent microstructural evolution patterns in both the horizontal and vertical directions, this study selects the horizontal direction for electron backscatter diffraction (EBSD) analysis to systematically characterize the grain size, morphology, and grain boundary misorientation in the weld zone. The inverse pole figure (IPF) maps and grain boundary (GB) maps of each joint region are shown in Figure 10a–d. The BM (Figure 10a) presents a typical rolled microstructure, with grains elongated along the transverse direction (TD) and compressed along the normal direction (ND), consistent with the metallographic observations in Figure 9(b_1_,b_2_). The HAZ (Figure 10b) exhibits a similar slender grain morphology to BM, but the grain size is larger than that of BM. This is because the grains undergo coarsening under the effect of thermal cycling. The TMAZ (Figure 10c) presents a dual-state structure where elongated grains in the TD direction coexist with substructured fine grains. The elongated grains are caused by the friction of the stirring needle during the FSW process and are characterized mainly by high-angle grain boundaries (HAGB, θ > 15°). The formation of fine sub-grains is closely related to plastic deformation and mainly consists of low-angle grain boundaries (LAGBs, 0 < θ < 15°). By comparison, the SZ (Figure 10d) exhibits a homogeneous equiaxed fine-grained structure without elongated grains, which is different from other welding areas. Based on the EBSD orientation data (Figure 10) and using the linear intercept method [37], the grain size distribution is quantified as shown in Figure 11a. The average grain sizes of the BM and HAZ are 9.72 μm and 11.77 μm, respectively. The increase in grain size in the HAZ confirms the grain coarsening caused by the welding thermal cycle, which aligns with the observations in Figure 10b. Due to the mixed structure containing both HAGB-defined large grains and LAGB-defined sub-grains, the average grain size in the TMAZ decreases to 6.27 μm. In contrast, the average grain size in the SZ is only 1.3 μm, approximately 13.4% of that in the BM. These results indicate that, compared to the other regions, the SZ achieves significant grain refinement due to complete DRX.

The kernel average misorientation (KAM) maps in Figure 10e–h quantitatively characterize the degree of local lattice distortion in each region, with their average KAM values summarized in Figure 11a. The KAM value reflects the crystallographic orientation deviation between adjacent measurement points and is directly related to localized strain induced by high dislocation density [38]. Analysis indicates that the HAZ (Figure 10f) exhibits more extensive locally deformed zones (green areas) compared to the BM (Figure 10e), with the average KAM value increasing from 0.43° to 0.52°. This is likely attributed to residual dislocations introduced by partial grain coarsening and recovery processes induced by the thermal cycle. In contrast, the BM grain structure remains stable with low dislocation density and is largely unaffected. In the TMAZ (Figure 10g), significant heat input and intense plastic flow result in high dislocation density and severe lattice distortion within the grains. This leads to an increase in highly localized deformation zones and a marked rise in the average KAM value to 0.6°. Notably, the SZ (Figure 10h) shows the lowest average KAM value of 0.5° and a reduction in locally deformed zones, yet it remains higher than that of the BM. This phenomenon may be associated with the dislocation accumulation mechanism involved in the formation of new grains.

Figure 10i–l and Figure 11b respectively show the distribution statistics of grain boundary orientation differences and the LAGBs/HAGBs ratio in the welding zone, further revealing the evolution mechanism of the microstructure. In the BM, the proportion of HAGBs is significantly higher than that of LAGBs, at 93.21% and 6.79% respectively. This is due to the minimal thermal influence, where the annealed, fully developed grain structure dominates, characterized by large grain boundaries. Compared to the BM, the proportion of LAGBs in the HAZ increases to 14.9% (HAGBs: 85.1%). This increase results from thermal stresses introduced by the welding thermal cycle, which generate dislocations and consequently form more LAGBs. Compared to the BM and HAZ, the TMAZ exhibits a significantly higher proportion of LAGBs (20.54%) and a lower proportion of HAGBs (79.46%) due to the combined effects of heat input and material plastic deformation. This leads to pronounced dislocation multiplication, grain fragmentation, and the formation of substructures. In the SZ, the proportions of LAGBs and HAGBs are 13.68% and 86.32%, respectively. The formation of fine equiaxed grains is closely related to DRX. However, rapid cooling inhibits the complete progression of recrystallization, resulting in a slightly higher residual proportion of LAGBs than BM.

To sum up, based on the grain structure, KAM distribution and grain boundary characteristics, it can be known that the microstructure of HAZ and BM is highly similar. This is primarily because the HAZ is less affected by the plastic deformation induced by the stirring tool [39]. In contrast, the TMAZ and SZ undergo significant changes in grain size, misorientation distribution, and dislocation density due to thermo-mechanical coupling effects, leading to a fundamental restructuring of their microstructures.

Based on the analysis of Figure 9, Figure 10 and Figure 11, it can be known that the welded joint mainly includes coarse grains elongated along the deformation direction in BM and HAZ, as well as refined grains in TMAZ and SZ. This indicates that significant thermomechanical coupling occurs between the stirring tool and the workpiece during FSW, leading to distinct temperature gradients and stress distribution differences within the weld zone. Among them, the SZ develops a typical fine equiaxed grain structure, accompanied by a high proportion of HAGBs and a relatively low dislocation density. This suggests that dynamic recovery (DRV) and DRX take place in this region under thermomechanical action, resulting in dislocation reorganization and grain refinement [40].

**Figure 12:**
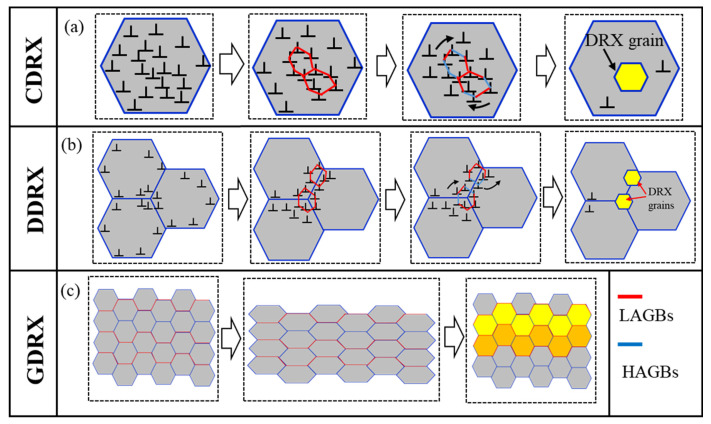
Schematic diagram of dynamic recrystallization mechanism: (**a**) CDRX, (**b**) DDRX and (**c**) GDRX processes.

DRV and DRX, as important mechanisms in the high-temperature deformation process, can effectively release the internal stress generated during processing and are commonly found in hot working processes such as hot rolling and extrusion. Al/Al-Li alloys are prone to DRV and DRX at FSW temperatures due to their high stacking fault energy and relatively low melting point [41]. Generally, DRX can be classified into three main types based on its nucleation and growth characteristics: (i) continuous dynamic recrystallization (CDRX), (ii) discontinuous dynamic recrystallization (DDRX), and (iii) geometric dynamic recrystallization (GDRX). Among them, DDRX is commonly found in low-stack-layer-fault-energy materials, while CDRX and GDRX are more prevalent in high-stack-layer-fault-energy materials. The schematic diagrams of these three types of DRX mechanisms are shown in Figure 12. CDRX (Figure 12a) promotes the annihilation and rearrangement of dislocations through the DRV process, leading to the formation of subgrains with low-angle grain boundaries (LAGBs) within the original grains. During subsequent deformation, to accommodate plastic flow and reduce local strain energy, these subgrains undergo crystallographic rotation, causing the misorientation between adjacent subgrains to progressively increase with strain accumulation. When the misorientation exceeds a critical value, forming high-angle grain boundaries (HAGBs), new grains are ultimately generated through the migration and coalescence of subgrain boundaries. In DDRX (Figure 12b), DRV at the initial grain boundaries promotes the nucleation of new grains. Under low-strain conditions, dislocation scaling and cross-sliding occur near the original HAGBs due to stress concentration [42], forming dislocation cells. As strain increases, the annihilation and reorganization of dislocation cells cause the rotation of subgrains containing LAGBs, which gradually transform into DRX grains containing HAGBs. In contrast, the mechanism of GDRX is relatively straightforward, as illustrated in Figure 12c. During deformation, the original grain boundaries are sheared and compressed, bringing HAGBs into direct contact with each other. This leads to the fragmentation of coarse grains into multiple fine grains with similar orientations.

Previous studies have shown that most metallic materials generally undergo DRX during the FSW process, and the dominant mechanism is closely related to the stacking fault energy of the materials [43]. For instance, in Mg-Y-Zn alloys with low stacking fault energy, DDRX is dominant [44]. However, for Al/Al-Li alloys with high stacking fault energy, the DRX process primarily follows an evolution path of DRV → CDRX/GDRX, making DDRX unlikely to occur. Therefore, it is generally believed that the dominant DRX mechanism in Al/Al-Li alloys treated with FSW is CDRX or GDRX [45]. Notably, temperature distribution across different weld zones during FSW varies due to thermal gradients. The welding heat input is directly affected by process parameters such as *w*, *v* and *h*, etc. For instance, when FSW is carried out on 7055 aluminum alloy at *w* = 350 rpm and *v* = 60 mm/min, the SZ temperature can reach over 450 °C, while the TMAZ and HAZ temperatures exceed 290 °C and 280 °C, respectively [46]. Similarly, when welding 2219 aluminum alloy at *w* = 400 rpm and *v* = 100 mm/min, temperatures in the SZ and HAZ reach 508 °C and 429 °C, respectively [47]. Based on these studies, it can be inferred that under the optimal parameters used in this experiment (400 rpm, 60 mm/min, and 0.21 mm), the SZ subjected to severe plastic deformation may have reached a high temperature of at least 350 °C, which is sufficient to induce complete DRX. The TMAZ also experienced considerable plastic deformation and frictional heating, but its deformation degree and heat input were lower than those in the SZ. Consequently, the relatively insufficient temperature in the TMAZ likely resulted in incomplete DRX. Compared to the SZ and TMAZ, the HAZ undergoes minimal plastic deformation and limited heat input, leading to only slight recrystallization or even the absence of DRX.

The EBSD characterization results (Figure 11a,b) further support the above inferences. The SZ exhibits the finest average grain size (1.3 μm), the highest proportion of HAGBs (86.32%), and the lowest average KAM value (0.5°), indicating that this region displays typical CDRX characteristics under the combined effect of intense mechanical action from the tool pin and frictional heat [48]. In contrast, the average grain size (6.27 μm), average KAM value (0.6°), and HAGB ratio (79.46%) of TMAZ. Due to relatively lower plastic deformation and heat input, DRX in the TMAZ is incomplete, and its recrystallization behavior is predominantly governed by the GDRX mechanism [49]. Meanwhile, the microstructure of HAZ is relatively close to that of BM, indicating that the plastic deformation in this area is small during the welding process, and the DRX phenomenon is not obvious. In summary, during the FSW process, the frictional heat generated by the stirring tool and the plastic deformation of the material have significant differences in their effects on different regions, which leads to SZ, TMAZ, and HAZ, respectively, demonstrating varying degrees of DRX behavior and mechanism characteristics.

#### 3.4.3. Hardness

**Figure 13:**
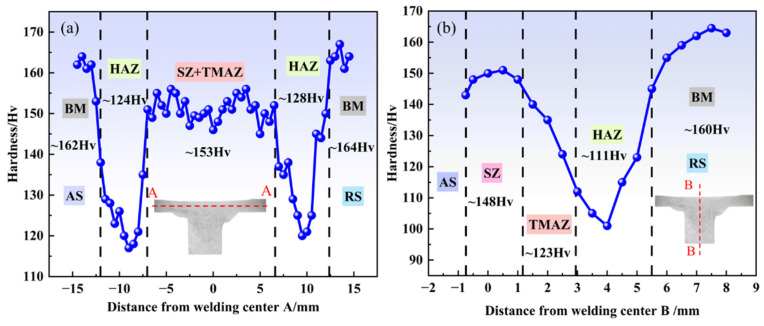
Microhardness distribution of T-joint under the optimal parameters: hardness along (**a**) the skin, and (**b**) the stringer.

According to ASTM E384 standard, the microhardness distribution of each joint region (SZ, TMAZ, HAZ, and BM) was measured under a dwell time of 10 s, as shown in Figure 13. The hardness profiles along the horizontal direction (A-A) and the vertical direction (B-B) exhibit typical “W” and “N” type distributions, respectively. The SZ and TMAZ show relatively high microhardness, with an average value of approximately 153 Hv, which is primarily attributed to the combined strengthening effects of high dislocation density in the TMAZ and grain refinement in the SZ. In contrast, the lower hardness in the HAZ (~111–128 Hv) makes it a mechanically weak zone, consistent with the fracture location observed in the previous tensile tests. The hardness of the BM remains within the range of ~160–164 Hv. Furthermore, the figure indicates a significant negative correlation between weld hardness and grain size (see Figure 11), suggesting that grain-refinement strengthening is a key factor in enhancing the hardness of the T-joint [50], and DRX is the main mechanism responsible for grain refinement [51]. During FSW, intense plastic deformation disrupts the grains in the affected regions, increasing the dislocation density of the original grains and promoting the occurrence of a stronger DRX phenomenon. Dislocations continuously reorganize and accumulate at LAGBs, increasing the misorientation between adjacent subgrains. As plastic deformation proceeds, LAGBs gradually transform into HAGBs, eventually leading to the formation of finer new grains. Thus, grain size is typically determined by the coupled effects of temperature and the degree of plastic deformation. According to the Formula (5) mentioned by Scholar Liu, it represents the above relationship [52]:

(5) $$d^{2}=d_{0}^{2}+k_{0}t\;\exp(-\frac{Q}{\mathrm{RT}})$$

Among them, *d* is the final grain size, *d*_0_ is the initial grain size, *k*_0_ is a constant, *Q* is the activation energy of grain boundary migration, *t* is time, *R* is the gas constant, and *T* is temperature. This is highly consistent with the DRX behavior in each area of the previous joint. In the SZ with the most sufficient thermal–mechanical conditions, the grains are completely refined, making its hardness reach 94.4% of BM. This provides further theoretical confirmation that grain refinement is the primary reason the joint hardness approaches the level of the BM.

#### 3.4.4. Tensile Properties

Tensile tests show that the optimized T-joint achieves a UTS of 377 MPa, and all specimens fractured in the HAZ on the advancing side (AS) of the 7055-aluminum alloy, with the crack terminating in the SZ (see Figure 3). The joint attains its optimal comprehensive performance at the medium level of process parameters (coded level 0). When the process parameters deviate toward the low (−1) or high (+1) level, the welding heat input increases significantly, causing excessive deformation of the BM and grain coarsening in the SZ, which leads to an overall reduction in the strength of this region. Further investigation reveals that the effective bonding of materials is governed by the synergistic effect of heat input and strain rate. During FSW, the relative velocity between the tool and the workpiece is higher on the AS than on the retreating side (RS), resulting in a notably higher frictional heat generation rate and plastic deformation dissipation energy on the AS. Consequently, the AS of the SZ experiences more intensive thermomechanical coupling, forming finer recrystallized grains and denser metallurgical bonding, which is the primary reason for the significant improvement in joint strength. However, the higher heat input on the AS also intensifies the overheating of the HAZ. As 7055 aluminum is a precipitation-strengthened alloy, the HAZ undergoes coarsening and dissolution of precipitates under the thermal cycle, leading to pronounced softening in this region. This makes the HAZ the weakest link of the joint, where micro-cracks preferentially generate. Subsequently, the crack propagates along the direction of maximum shear stress. Due to the geometric characteristics of the T-joint, the shoulder diameter of the tool is larger than the width of the stringer, creating a significant geometric stress concentration at the transition between the SZ and the stringer. This stress concentration effect causes the crack propagation path to deflect and eventually penetrate the SZ. Although the optimized SZ possesses a refined grain structure and high strength, its fracture toughness is still insufficient to suppress unstable crack propagation, resulting in a joint strength that reaches only 74.1% of the BM under the optimal parameters.

#### 3.4.5. Fracture Analysis

**Figure 14:**
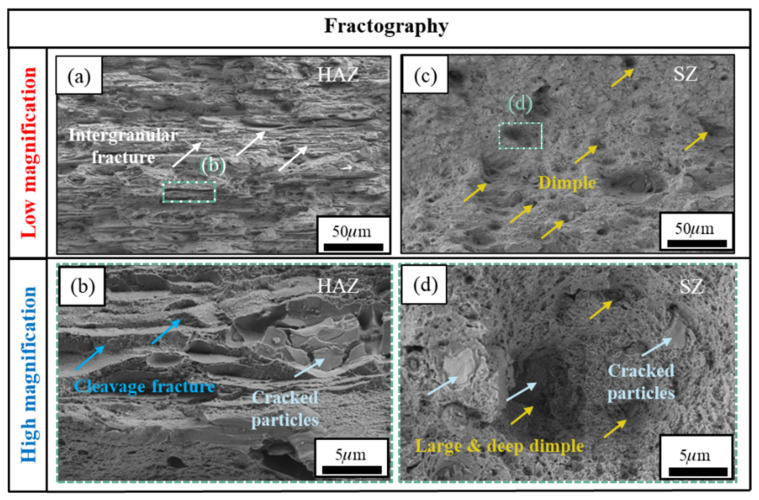
SEM micrographs of the fracture surfaces of T-joints: (**a**,**b**) HAZ and (**c**,**d**) SZ. (**c**,**d**) are magnified views of the green dashed boxes in (**a**,**b**), respectively.

**Figure 15:**
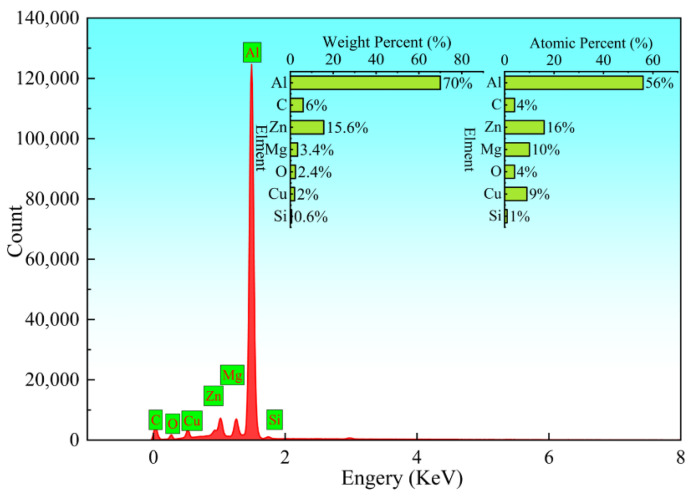
Element distribution mapping of EDS scanning in the weld fracture area.

To further reveal the failure mechanism of the T-joint, the fracture surfaces of the tensile specimens under the optimal parameters were systematically characterized by scanning electron microscopy (SEM), with typical fractographic features shown in Figure 14. Fractographic analysis indicates that the crack initiated in the HAZ, which exhibits typical intergranular fracture characteristics (Figure 14a). This can be attributed to the dissolution and coarsening of precipitates under the welding thermal cycle, leading to the formation of precipitate-free zones (PFZs) adjacent to the grain boundaries. Acting as local soft zones, the PFZs undergo plastic flow under stress, causing stress concentration at the neighboring grain boundaries, which ultimately weakens the boundaries and promotes intergranular separation. Furthermore, cleavage steps observed in this region (Figure 14b) further confirm the typical brittle fracture mode of the HAZ. In stark contrast, the fracture surface of the SZ is dominated by uniformly distributed equiaxed dimples (Figure 14c). At higher magnification, the dimples appear relatively deep, and there are micrometer-scale broken second-phase particles at the bottom (Figure 14d). These features fully demonstrate that the fracture mechanism in the SZ of the joint under optimal parameters is ductile fracture, which is closely related to the behavior of micro-pore aggregation and growth during the plastic deformation of the material. Additionally, to further investigate the influence of interfacial elements on the fracture behavior, energy-dispersive spectroscopy (EDS) was employed to analyze the elemental composition and distribution in the fractured regions (see Figure 15). The analysis revealed significant proportions of Al, Zn, Mg, O, and Cu elements in the fracture zone, indicating the potential formation of interfacial layers such as alumina (Al_2_O_3_), copper oxide (CuO), and magnesium oxide (MgO). Such oxides may enhance joint strength through mechanisms such as pinning dislocations or deflecting cracks [53], which aligns with the peak strength observed in the SZ during tensile tests, as shown in Figure 3 and Table 4. However, it should be noted that Al-Zn-Mg-Cu system alloys are known to be susceptible to the formation of brittle intermetallic phases under high heat input conditions. Although the specific identification of these phases (e.g., Al_3_Mg_2_or MgZn_2_) requires further high-resolution characterization techniques such as TEM, the observed enrichment of Al, Zn, and Mg in the fracture zone (Figure 15) suggests that solute segregation and potential precipitation of coarse equilibrium phases may have occurred. These microstructural features, combined with the stress concentration effect, could contribute to the preferential crack initiation observed in the HAZ. Moreover, abnormal enrichment of carbon (C) element was detected on the fracture surface. The source of this C remains unclear; possible origins include surface contaminants, residual lubricants, or environmental exposure during sample preparation. Further investigation is needed to clarify the origin and potential role of carbon in the fracture process.

## 4. Conclusions

Based on the BBD method, this study established the response surface model and conducted systematic research on the FSW T-joint of Al/Al-Li alloy, focusing on revealing the formation essence of the joint performance from the perspective of microstructure evolution and strengthening mechanism. Through multiple sets of process parameter combinations, welded joints free from macroscopic defects were successfully fabricated. By integrating microstructural and crystallographic analysis, the evolution laws of different zones under thermo-mechanical coupling were clarified. The main conclusions are as follows:

(1) The BBD method was used to construct the response surface model. Its accuracy and reliability were validated through ANOVA, normal probability plots of residuals, and the good agreement between the actual and predicted values. It can be known from the perturbation curve that *v* had the most significant influence on UTS and WNH, followed by *h*, and *w* has the least influence. Further analysis via 3D response surface plots and contour plots revealed that for both UTS and WNH, the interaction between *w* and *v* was the most significant, followed by that between *v* and *h*, while the interaction between *w* and *h* was the weakest. Model optimization yielded the optimal process parameters: *w* = 400 rpm, *v* = 60 mm/min, and *h* = 0.21 mm. Under these conditions, the joints of UTS and WNH reached 377 MPa and 153 Hv, respectively, corresponding to 74.1% and 94.4% of the BM.

(2) Microstructural analysis demonstrated that, despite compositional differences between the skin and stringer in the BM, the grain evolution patterns in each region are consistent. From the BM through the HAZ and TMAZ to the SZ, grains underwent a continuous evolution sequence of coarsening, partial recrystallization, and finally complete DRX. Simultaneously, the second-phase particles exhibited a coordinated evolution characterized by refinement and increasingly uniform distribution.

(3) Crystallographic analysis indicated that the HAZ microstructure resembled that of the BM, while the TMAZ and SZ underwent significant reconstruction due to thermo-mechanical coupling. The SZ experienced complete DRX under intense plastic deformation and high frictional heat, resulting in significant grain refinement and a relatively low dislocation density, with CDRX being the dominant mechanism. In contrast, the TMAZ, subjected to lower heat input and limited deformation, was governed primarily by the GDRX mechanism.

(4) The joint hardness exhibited typical “W” and “N” type distributions along the horizontal and vertical directions, respectively, with grain refinement being the primary strengthening mechanism. However, the thermal cycle caused coarsening and dissolution of strengthening precipitates in the HAZ, forming a softened zone that initiated intergranular brittle fracture. Meanwhile, high heat input promoted the precipitation of brittle compounds such as Al_3_Mg_2_ and MgZn_2_, accelerating crack initiation. Although interfacial oxides (e.g., Al_2_O_3_) could provide dislocation pinning strengthening, cracks still propagate under the drive of geometric stress concentration and penetrate SZ, which is mainly characterized by ductile fracture.

In summary, the FSW parameters optimized via RSM can significantly enhance the hardness and wear resistance of T-joints, providing an effective process solution to address fretting wear issues in critical structures such as wing stringer–skin connections. This study clearly reveals that for age-hardening alloys, HAZ softening remains the primary cause of joint mechanical property degradation. The key challenge for improving overall joint performance lies in effectively mitigating the strength loss of the HAZ. Therefore, future work will focus on thermal control strategies such as the development of FSW process cooling and low heat input tools. At the same time, transmission electron microscopy (TEM) will be employed to further investigate the nanoscale precipitation behavior, particularly the evolution of strengthening precipitates (e.g., η’ phases) in the HAZ and the reprecipitation mechanisms in the SZ. This will help clarify the microstructural origins of HAZ softening and fracture behavior. These investigations will complement the current EBSD and SEM analyses, establishing a more comprehensive understanding of the microstructure properties relationships in T-joints.

## Figures and Tables

**Table 1:** Chemical composition of 7055-T6 Al and 2195-T6 Al-Li alloys (mass fraction, %).

Materials	Zn	Mg	Cu	Zr	Fe	Mn	Si	Ti	Cr	Li	Ag	Al
7055-T6	7.9	2.1	2.3	0.05	0.15	0.05	0.1	0.06	0.01	-	-	Bal
2195-T6	0.12	0.42	3.78	0.08	0.06	0.11	0.05	0.02	-	0.87	0.28	Bal

**Table 2:** Room temperature properties of 7055-T6 and 2195-T6 alloys.

Materials	Transverse Tensile Strength/MPa	Longitudinal Tensile Strength/MPa	Elongation/%	Melting Point/°C
7055-T6	509	411	13.9	590
2195-T6	568	496	11.2	540

**Table 3:** Limits of the working process parameters.

Parameters	Notations	Units	Level		
			−1	0	1
Rotational Speed	*w*	rpm	300	400	500
Welding Speed	*v*	mm/min	50	60	70
Plunge depth	*h*	mm	0.19	0.21	0.23

**Table 4:** The design matrix has actual and coded values and their respective outputs.

Experimental Group	Factors			UTS (MPa)	WNH (Hv)
	*w* (rpm)	*v* (mm/min)	*h* (mm)		
1	300	50	0.21	155 ± 1.41	116.3 ± 3.25
2	500	50	0.21	332 ± 2.83	151.8 ± 0.28
3	300	70	0.21	239 ± 1.41	138.7 ± 0.99
4	500	70	0.21	128 ± 2.84	112 ± 2.82
5	300	60	0.19	129 ± 2.83	112.9 ± 0.14
6	500	60	0.19	168 ± 2.81	121 ± 1.41
7	300	60	0.23	177 ± 2.83	124 ± 0.42
8	500	60	0.23	192 ± 1.43	126.7 ± 2.82
9	400	50	0.19	182 ± 2.86	124 ± 0.57
10	400	70	0.19	161 ± 2.79	120.4 ± 0.85
11	400	50	0.23	251 ± 4.24	139.9 ± 0.71
12	400	70	0.23	166 ± 4.24	120.5 ± 1.41
13	400	60	0.21	372 ± 2.83	151 ± 0.57
14	400	60	0.21	375 ± 2.83	152.4 ± 0.42
15	400	60	0.21	374 ± 4.24	152.7 ± 2.82
16	400	60	0.21	376 ± 1.41	153 ± 1.41
17	400	60	0.21	377 ± 1.41	153 ± 3.15

**Table 5:** Statistical table of ANOVA data of UTS.

Source	Sum of Squares	df	Mean Square	F-Value	*p*-Value	
Model	150,500	9	16,723.30	450.59	<0.0001	significant
A-Rotational speed	1800.00	1	1800.00	48.5	0.0002	
B-Welding speed	6384.50	1	6384.50	172.02	<0.0001	
C-Plunge depth	2664.50	1	2664.50	71.79	<0.0001	
AB	20,736	1	20,736.00	588.71	<0.0001	
AC	144.00	1	144.00	3.88	0.0895	
BC	1024.00	1	1024.00	27.59	0.0012	
A^2^	33,802.78	1	33,802.78	910.78	<0.0001	
B^2^	18,396.67	1	18,396.67	495.68	<0.0001	
C^2^	53,859.41	1	53,859.41	1451.18	<0.0001	
Residual	259.80	7	37.11			
Lack of Fit	43.00	3	14.33	0.2645	0.8484	not significant
Pure Error	216.80	4	52.40			
Cor Total	150,800	16				
R^2^	0.99					
R^2^_adj_	0.95					
R^2^_pred_	0.94					
Adeq Precision	51.78					

**Table 6:** Statistical table of ANOVA data of WNH.

Source	Sum of Squares	df	Mean Square	F-Value	*p*-Value	
Model	4094.23	9	454.91	438.14	<0.0001	significant
A-Rotational speed	48.02	1	48.02	46.25	0.0003	
B-Welding speed	204.02	1	204.02	196.49	<0.0001	
C-Plunge depth	134.48	1	134.48	129.52	<0.0001	
AB	967.21	1	967.21	931.55	<0.0001	
AC	7.29	1	7.29	7.02	0.0330	
BC	62.41	1	62.41	60.11	0.0001	
A^2^	811.76	1	811.76	781.83	<0.0001	
B^2^	328.66	1	328.66	316.54	<0.0001	
C^2^	1272.58	1	1272.58	1225.66	<0.0001	
Residual	7.26	7	1.038			
Lack of Fit	4.5	3	1.5	2.17	0.2345	not significant
Pure Error	2.76	4	0.692			
Cor Total	4101.5	16				
R^2^	0.99					
R^2^_adj_	0.97					
R^2^_pred_	0.96					
Adeq Precision	52.72					

**Table 7:** Predicted optimized FSW results based on various criteria for UTS and WNH.

No	*w* (rpm)	*v* (mm/min)	*h* (mm)	UTS (MPa)	WNH (Hv)	Desirability	
1	431	57	0.21	370	153	1	Selected
2	453	55	0.21	363	153	1	No
3	375	54	0.22	341	150	1	No

## Data Availability

The original contributions presented in this study are included in the article. Further inquiries can be directed to the corresponding author.

## Nomenclature

FSW	Friction stir welding	BM	Base material
RSM	Response surface method	TMAZ	Thermomechanical affected zone
*w*	Rotational speed	HAZ	Heat-affected zone
*v*	Welding speed	SZ	Stirring zone
*h*	Plunge depth	BBD	Box–Behnken design
UTS	Ultimate tensile strength	PMM	Parametric mathematical model
WNH	Weld nugget hardness	ANOVA	Analysis of Variance
IPF	Inverse pole figure	DRV	Dynamic recovery
GB	Grain boundary	DRX	Dynamic recrystallization
HAGB	High-angle grain boundaries	CDRX	Continuous dynamic recrystallization
LAGB	Low-angle grain boundaries	DDRX	Discontinuous dynamic recrystallization
KAM	Kernel Average Misorientation	GDRX	Geometric dynamic recrystallization
